# The heterogeneity of genomic alterations, metastatic patterns and immune microenvironment in metastatic ovarian cancer originating from colorectal cancer

**DOI:** 10.3389/fimmu.2025.1593439

**Published:** 2025-06-11

**Authors:** Chao Chen, Jian Wang, Binjie Sun, Yuyan Zheng, Xiaoxu Ge, Zhiyuan Gong, Haochen Gu, Zhiwei Zhang, Akao Zhu, Yingkuan Shao, Yeting Hu, Lijia Ma, Yini Li, Kefeng Ding, Da Wang, Lifeng Sun

**Affiliations:** ^1^ Department of Colorectal Surgery and Oncology, Key Laboratory of Cancer Prevention and Intervention, Ministry of Education, the Second Affiliated Hospital, Zhejiang University School of Medicine, Hangzhou, Zhejiang, China; ^2^ Department of Cancer Institute, The Second Affiliated Hospital, Zhejiang University School of Medicine, Hangzhou, Zhejiang, China; ^3^ School of Computer Science, Fudan University, Shanghai, China; ^4^ School of Life Sciences, Westlake University, Hangzhou, China; ^5^ Zhejiang University Cancer Center, Hangzhou, Zhejiang, China

**Keywords:** ovarian metastases, colorectal cancer, whole exome sequencing, genetic alterations, phylogenetic tree analysis, metastatic pattern, neoantigen

## Abstract

**Purpose:**

The ovarian metastases originating from colorectal cancer (CRCOM) develops rapidly and lethally. Previously, the genetic alterations and metastatic pathway in CRCOM were not well understood. The aim of this study is to explore the special molecular phenotype and dissemination patterns of CRCOM.

**Methods:**

The whole-exome sequencing (WES) was performed on 65 matched tissue samples from 11 CRCOM patients, including 11 primary colorectal cancer (CRC) with 11 matched normal tissues, and 43 multi-site metastases (including 15 CRCOMs and 4 patients had bilateral ovarian metastases (OMs). Genetic landscape, neoantigens, tumor clonal origin and spread of CRCOMs were analyzed. TCGA-COAD dataset combined with our data were used for survival analysis and validation of the findings.

**Results:**

There was significant intertumoral heterogeneity among patients with CRCOM and intra-tumoral heterogeneity among multiorgan metastases. 19 genes were inferred as the potential driver genes of CRCOM. USP7 and RPA1 were HRD-related mutations and potential to serve as predictive biomarkers in OM. The putative neoantigen number of the primary CRC and OM varies widely among patients. The OM showed an immune desert state, extremely deficient in each subtype of immune cells. According to COSMIC signatures features, the CRCOM patients were divided into two groups, which are different in overall survival (OS) (median OS, 720 days vs 360 days, *P =* 0.074) and genetic alterations. Two metastatic patterns of CRCOM were summarized, which were primary CRC to OM, and metastases to metastases (including lymph node metastases (LNM) to OM, peritoneal metastases (PM) to OM, and other metastases to OM). Interestingly, the sources of bilateral OM might be different in the two patients.

**Conclusion:**

This study presents a better understanding the heterogeneity of the genetic characterizations and metastatic pattern in CRCOM. The subtypes of CRCOM with USP7 mutation, more copy number alterations, lower neoantigens, and immunoscore have a worse prognosis.

## Highlights

- CRCOM was classified into two subtypes, indicating the heterogeneity of CRCOM patients. The subtypes with USP7 mutation and more copy number alterations had a worse prognosis, and lower neoantigen numbers and immunoscore.
- The metastasis pathways of CRCOMs can be classified into two categories: one pattern is direct metastasis of the primary lesions to the ovary; Another pattern may be from other metastatic sites to the ovary.
- It was discovered that the metastatic pathways of bilateral ovarian metastases of colorectal cancer may be different.

## 1 Introduction

Colorectal cancer (CRC) is one of the digestive system malignant tumors with the highest incidence in the population, and its mortality rate ranks the top three among all malignant tumors (1). With the development of detection and treatment of CRC, the survival time of CRC patients has been prolonged, however, distant metastasis is still a big challenge (2). About 2-9% of female CRC patients were combined with ovarian metastases (OM) at initial diagnosis, as well as 0.4-7% of female CRC patients with metachronous OM (3–6). The incidence of colorectal cancer with ovarian metastases (CRCOM) has been rising in recent years due to the development of imaging techniques for metastatic colorectal cancer (6). OM often occurs in young female CRC patients (7), meanwhile, CRCOM is progressing rapidly and relatively resistant to chemotherapy (8–10). Compared with primary CRC and other distant metastases, there are fewer effective treatments for CRCOM due to the special molecular characteristics and unclear evolutionary relationship between OM and primary CRC (10, 11). Despite receiving active treatment with surgery, chemotherapy, and immunotherapy, the median overall survival time of patients with CRCOM was only 10.0 months (4, 7) (less than 30 months reported by CALGB 80405 (12), a large clinical trial of CRC patients with distant metastases). Given its potential impact on patient care, a better understanding of the special molecular phenotype and metastatic pathways of CRCOM could prolong the survival time and improve the quality of life among these patients.

Researchers have proposed various mechanisms in primary CRC metastasizing to distant organs. According to anatomy, regional lymph nodes (RLN) are the first step after cancer cells detach from the primary tumor and then distant metastasis (13–16), but a part of patients with CRCOM didn’t have lymph node metastases (LNM). Some studies have shown that OM originated from the implantation metastasis of primary CRC (10, 17). Primary CRC cells penetrate the serosal layer and fall off into the peritoneal cavity or ascites, eventually reaching the ovarian capsule through intestinal peristalsis and gravity, and then developing into OM (18). However, it was found that the infiltration depth of primary CRC did not reach the serosal layer and the metastases were located in the ovarian stroma rather than on the ovarian surface in some patients with CRCOM. In addition, although the metastases were large, the capsule was intact. Other scholars believed that peritoneal metastases (PM) were an important source of OM because the ovary and peritoneum have similar biological behaviors and most patients with CRCOM also experienced PM (19, 20).

With the progress of whole exon sequencing (WES), some researchers have illustrated that distant metastasis may be spread from one or more subclones in any cancer site, including primary cancer and metastatic cancer (21, 22), and suggested that genetic divergence and heterogeneity of metastatic cancer (23, 24). Cancer cells, tumor microenvironment, signaling pathways, and special molecules related to cancer metastasis constantly adjust and change to promote the invasion and growth of cancer cells (25–27). Thereafter, these cancer cells continue to evolve and acquire private mutations, thus metastasizing to other organs and forming metastases (28, 29). To date, most studies focused on the relationship between primary CRC and distant metastasis by using single pairing, for example, primary CRC paired with brain metastases or liver metastases. It remains lacking in the integrated metastatic evolution of multiple metastases from CRC, especially OM, which is associated with poorer prognosis relative to other organ metastases such as liver or lung metastasis.

In this study, we performed WES on 65 samples, including matched primary CRC, normal tissues, and multiorgan metastases, from 11 patients with CRCOM. We are the first to characterize the molecular phenotype and the clonal evolution pattern of CRC with OM using comprehensive genetic sequencing. The purpose of our study was to investigate the mysterious nature of CRCOM and identify the CRCOM with distinct molecular and clinical features that capture the clinical heterogeneity in CRCOM and could direct future therapy development.

## 2 Methods

### 2.1 Patients and specimens

The study protocol was reviewed and approved by the Ethics Committee of the Second Affiliated Hospital of Zhejiang University School of Medicine (SAHZU). We collected 65 tissue samples from 11 patients with the microsatellite-stable (MSS) CRC at SAHZU from 2016 to 2018. All the primary and metastatic tumors were collected from these patients, including 11 primary CRC and 11 matched normal tissues, 10 paracolic lymphonode metastases (LNM), 3 liver metastases (LM), 5 omentum metastases (OMM), 8 peritoneal metastases (PM), 1 spleen metastasis (SpM), 2 tumor deposits (TD) and 15 CRCOMs. Patients 1, 4, 8, and 10 had bilateral OM, while the remaining 7 patients had unilateral OM.

HE-stained sections from each sample were reviewed to confirm that the tumor specimen was histologically consistent with metastatic CRC (>40% tumor cells) and that the adjacent tissue specimen contained no tumor cells by two independent pathologies.

### 2.2 Whole exome sequencing

Genomic DNA from formalin-fixed paraffin-embedded (FFPE) samples was extracted using QIAamp DNA FFPE Tissue Kit (Qiagen), and fragmented by M220 Focused ultrasonicator (Covaris) into ~250 bp. The whole genome library was prepared using KAPA Hyper Prep Kit (KAPA Biosystems). Exome capture was performed using the Illumina Rapid Capture Extended Exome Kit (Illumina Inc.). Enriched libraries were sequenced using the Illumina HiSeq 2500 platform as paired 125-bp reads, to reach the mean coverage of ~80X for the normal control and ~250X for the tumor samples. Raw VCF data has been deposited in the Genome Sequence Archive in the National Genomics Data Center, China National Center for Bioinformation/Beijing Institute of Genomics, Chinese Academy of Sciences, under accession number GVM000406 (Project: PRJCA011872). The median depths of whole-exome sequencing coverage across all tumor and normal colon tissues were 219× (43× to 661×) and 223× (100× to 665×), respectively, both of which were deeper than those from the whole-exome dataset in TCGA-COAD (**Supplementary Data 6**).

### 2.3 Single nucleotide variation

Paired-end sequencing data from WES were aligned to the reference human genome (Homo_sapiens_assembly38.fasta) using the Burrows-Wheeler Aligner with default parameters(bwa-mem). Alignment results (BAM files) were further processed for de-duplication, base quality recalibration, and indel realignment using the Picard tools (http://picard.sourceforge.net/) and Genome Analysis Toolkit (GATK4.0). Point mutations were called using Mutect2. All variants (single nucleotide variants, SNVs) were annotated using the Ensembl Variant Effect Predictor v89 (https://www.ensembl.org/info/docs/tools/vep/) and ANNONAR (https://annovar.openbioinformatics.org/en/latest/) incorporating COSMIC v90, dbSNP build 146, Exome Aggregation Consortium (Exac03) and clinvar_20190305 annotations. For SNVs, we used maftools tools (R packages) to plot the summary of SNVs, which displays a number of variants in each sample as a stacked barplot and variant types as a boxplot summarized by Variant_Classification, and to draw a waterfall plot (Oncoplots).

### 2.4 Copy number alterations

Sequenza (v3.0.0 R packages) was used to call CNAs while considering both ploidy and cellularity. Briefly, we used BAM files from the WES data of each tumor and the paired normal samples as input to calculate the depth ratio, which was normalized based on both GC content bias and the data ratio. To acquire segmented copy numbers and estimate cellularity and ploidy. For each tumor sample, the copy numbers of segments were then divided by ploidy following log2 transformation. Copy number gains and losses were analyzed by GISTIC2.0. Among these gains and losses, amplifications were defined as four or more copies more than the ploidy, whereas deletions were defined as total deletion of the segment. Finally, CNA visualization was by Integrative Genomics Viewer (IGV) (http://www.broadinstitute.org/igv/).

### 2.5 Phylogenetic trees

The cancer cell fraction (CCF) of somatic mutations across all regions in each patient was estimated by PyClone (v0.13.0), a hierarchical Bayesian model incorporating local CNAs and SNVs. We also included mutations that were not located in exome regions to improve the sensitivity of the analysis.

Next, ClonEvol packages (R3.6) were used for phylogenetic inference from CCF subclones and the following visualization. Briefly, this tool first enumerates all trees independently for each sample and then tries to build a ‘consensus’ tree model that fits multiple samples from a single patient at once. We successfully obtained consensus models in 11 patients and constructed phylogenetic trees accordingly.

MEGA 11(Molecular Evolutionary Genetics Analysis)is an open-source software that integrates sequence alignment, sequence analysis, and phylogenetic tree construction (30).

### 2.6 Potential driver genes in CRCOM

MutSig2CV (31), dNdScv (32), and OncodriveCLUST (33) were used to generate potential driver genes. Of the three computational tools, dNdScv, MuSig2CV, and OncodriveCLUST are all based on mutation frequency; MutSig2CV was used to identify genes that were mutated more often than expected by chance given the background mutation processes. The dNdScv is a group of maximum-likelihood dN/dS methods designed to quantify selection in cancer and somatic evolution, and uses trinucleotide context-dependent substitution matrices to avoid common mutation biases affecting dN/dS. OncodriveCLUST is based on the fact that most of the variants in cancer-causing genes are enriched at a few specific loci (aka hot spots) and takes advantage of such positions to identify cancer genes. It could detect genes with a significant bias toward mutation clustering in specific protein regions using silent mutations as a background mutation model. Genes were deemed significant at a q-value of 0.1. Collectively, we used candidate genes identified in either method or merged them.

The unsupervised clustering was performed by using the hclust function (the agglomeration method is “ward. D2”) in R software (Version 4.0.2).

### 2.7 Putative neoantigens identification and prediction

The OptiType algorithm was utilized for HLA typing (34). Non-silent mutations were employed to create a list of mutant peptides, each approximately 9–11 amino acids long, with the altered residues represented in each position. NetMHCpan (v4.0) was then applied to predict the binding affinities of both the mutant and corresponding wild-type peptides to the patient’s germline HLA alleles (35). Neoantigen candidates were identified based on a predicted mutant peptide binding affinity of less than 500 nmol/L and a rank of less than 2.

### 2.8 Immunohistochemistry

Paraffin-embedded slides of primary CRC and OM were stained by labelling the CD3+ (BOSTER, No. PB0112), CD8+ (BOSTER, No. PB0235) T cells and CD20+ (BOSTER, No. PB0028) B cell with specific antibodies. All the slides were stained with hematoxylin and eosin (HE). The CD3+, CD8+, and CD20+ stained cells were executed by a pathologist. The hot spots with positive staining were obtained. Computer-assisted calculations of the density of the positively stained immune cells were performed using Image J software (National Institute of Health, Bethesda, MD, USA).

### 2.9 Statistical analysis

Statistical analyses were performed with SPSS 22.0 and GraphPad Prism software. Continuous variables were analyzed by the student’s t-test, one-way ANOVA, or two-way ANOVA test. Survival and univariate analysis were determined by Kaplan–Meier analysis, and statistical analysis was calculated with the log-rank test. All statistical analyses were two-sided, and P value <0.05 was considered statistically significant.

## 3 Result

### 3.1 Information of samples

**Figure 1:**
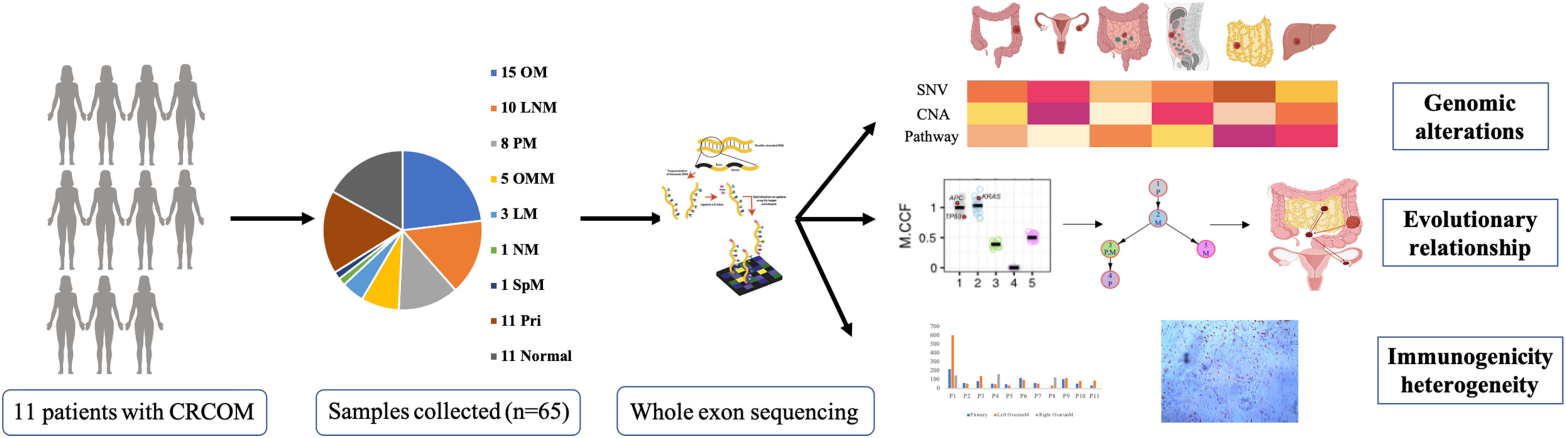
Study design. The flow chart showed the design and workflow of our research.

The workflow was presented in **Figure 1A**. 11 patients with CRCOM who underwent primary and metastatic surgery in our hospital were included in this study. A total of 65 patient-matched samples were collected, including 11 primary CRC and 11 matched normal tissues, 10 paracolic LNMs, 3 liver metastases (LM), 5 omentum metastases (OMM), 8 PMs, 1 spleen metastasis (SpM), 1 nodule metastasis (NM) and 15 OMs. Patient 1 (P1), P4, P8, and P10 had bilateral OM, while the remaining 7 patients had unilateral OM. The basic information on CRCOM patients and samples is shown in **Table 1**. The average age of our cohort was 46 years old (range 28-60). There were 4 cases of right colon cancer and 5 cases of left colon cancer, 2 cases of rectal cancer. Moreover, most of the patients presented with histologically confirmed adenocarcinoma whereas only one patient was presented with signet ring cell carcinoma. All patients are microsatellite stable (MSS). The median overall survival time of patients with CRCOM was 12 months.

**Table 1:** Clinical information of patients with CRCOM in our hospital.

Patient ID	Age	Tumor location	Pathology	Grade	DMR	OS	Time of OM	tumor size	Normal	Primary CRC	LNM	OM	PM	LM	OMM	Other metastasis	Samples number
P1	53	Rectum	Adenocarcinoma	Moderately	MSS	12	Synchronous	3*2*1.5	Yes	Yes	Yes	Bilateral	Yes	Yes	No		7
P2	43	Left colon	Adenocarcinoma	Moderately	MSS	12	Metachronous	left: 11*6*3.5 right: 11*8*6	Yes	Yes	Yes	Unilateral	Yes	Yes	No		6
P3	37	Right colon	Adenocarcinoma	Moderately	MSS	39	Synchronous	18*12*7	Yes	Yes	Yes	Unilateral	Yes	No	No	Spleen metastasis	6
P4	44	Left colon	Adenocarcinoma	Moderately	MSS	9	Synchronous	left: 8*5*5 right: 20*10*8	Yes	Yes	Yes	Bilateral	No	No	No		5
P5	42	Right colon	Adenocarcinoma	Moderately	MSS	24	Synchronous	6.5*4.5*4.2	Yes	Yes	Yes	Unilateral	Yes	No	No		5
P6	72	Left colon	Adenocarcinoma	Moderately	MSS	9	Synchronous	16*12.5*7	Yes	Yes	Yes	Unilateral	Yes	Yes	Yes		7
P7	28	Left colon	Signet-ring cell carcinoma	Signet-ring cell carcinoma	MSS	16	Metachronous	10*7*5	Yes	Yes	Yes	Unilateral	Yes	No	Yes		6
P8	47	Rectum	Adenocarcinoma	Moderately	MSS	20	Synchronous	left: 3*2.2*1.5 right: 4*4*1.7	Yes	Yes	Yes	Bilateral	No	No	Yes		6
P9	52	Right colon	Adenocarcinoma	Moderately to poorly	MSS	40	Metachronous	10*8*5	Yes	Yes	Yes	Unilateral	Yes	No	Yes		6
P10	60	Left colon	Adenocarcinoma	Moderately to poorly	MSS	11	Metachronous	left: 6*7*6 right: 5*6*4	Yes	Yes	No	Bilateral	Yes	No	Yes		6
P11	28	Right colon	Adenocarcinoma	Moderately	MSS	6	Synchronous	20*16*9	Yes	Yes	Yes	Unilateral	No	No	No	Tumors deposits	5
Summary									11	11	10	15	8	3	5	2	65

### 3.2 Genomic alterations across CRCOM

**Figure 2:**
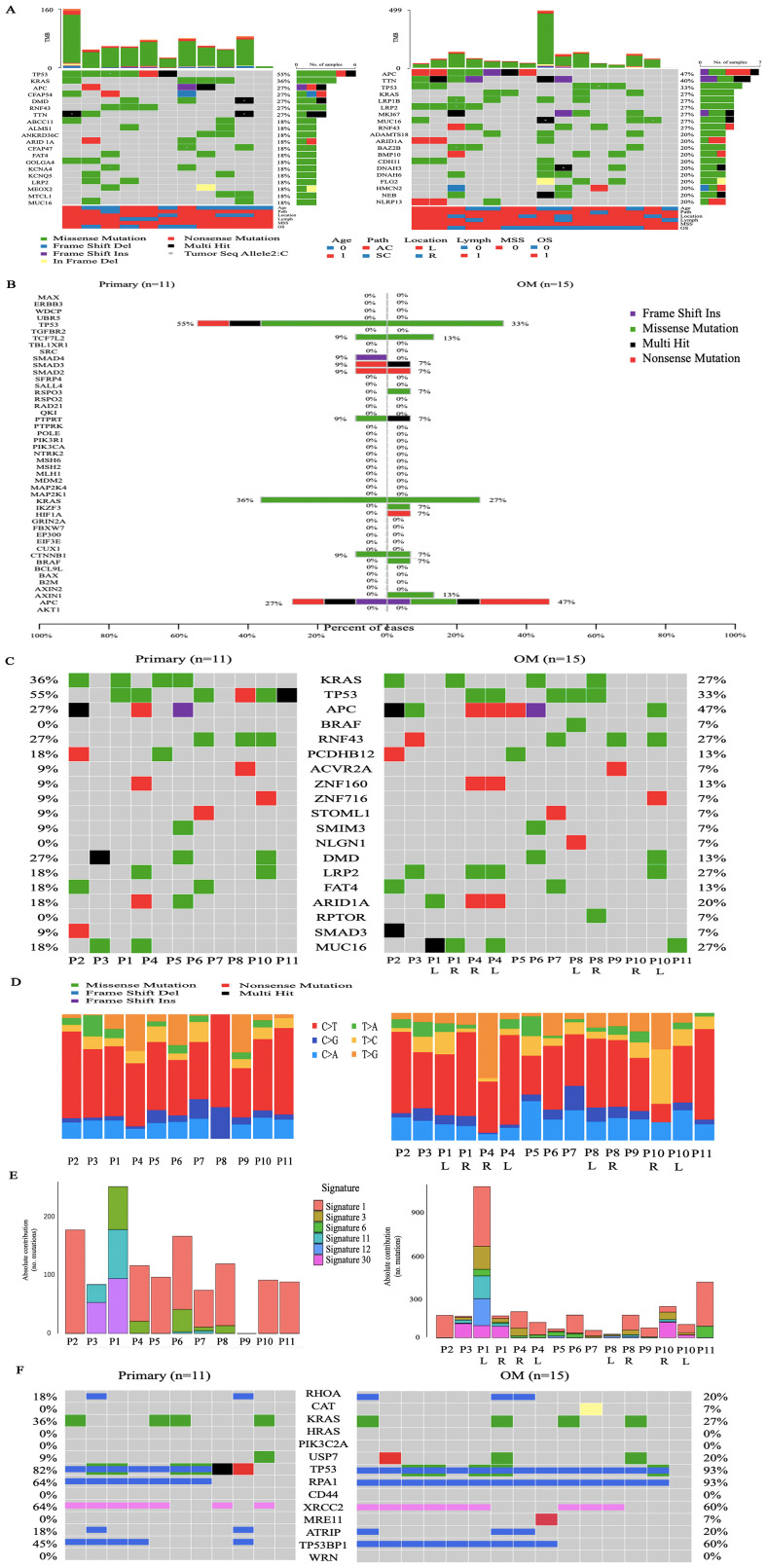
Genomic alterations across CRCOM. **(A)** The top 20 alteration spectrums of primary CRC and OM, the demographic and clinical information of the 11 patients was shown in the bottom. **(B)** The mutation of known 47 CRC driver genes in primary CRC and OM. Driver gene identification is based on the COSMIC Cancer Gene Census. **(C)** The list of 19 potential driver genes was identified by using OncodriveCLUST, MutSigCV2.0 and dNd Scv. **(D)** The single-nucleotide variations (SNVs) in primary CRC and OM. **(E)** The contributions of various signatures in primary CRC and OM based on the COSMIC Mutational Signatures database. **(F)** The mutations of DSB-related genes in primary CRC and OM.

We performed whole-exome sequencing (WES) and the average sequencing depth of tumor and normal samples was 145x (range 49x-289x) (**Supplementary Data 1**). The mean tumor mutation burden (TMB) for primary tumors and ovarian metastases was 10.73 and 6.46 mutations per megabase, respectively (**Supplementary Figure 1**, **Supplementary Data 2**). We calculated the mutated genes of all samples and the top 20 alteration spectrums of primary CRC and OM are shown in **Figure 2A** (**Supplementary Data 3**). Among the top 20 genes with the highest alteration rates, TP53 (55%), KRAS (36%), and APC (27%) were the 3 genes with the highest alteration rates in CRC primary CRC. APC (47%), TTN (40%), and TP53 (33%) were the 3 genes with the highest alteration rates in OM. We also marked the known 47 CRC driver genes based on the list from the COSMIC Cancer Gene Census in primary CRC and OM, respectively. CRC driver genes with high alteration rates in primary CRC and ovarian metastasis, include APC (27% *vs* 47%), KRAS (36% *vs* 27%), and TP53 (55% *vs* 33%). AXIN1, BRAF, HIF1A, KZF3, and RSPO3 alternated only in OM, and SMAD4 mutated only in primary CRC (**Figure 2B**). We used three different tools (OncodriveCLUST, MutSigCV2.0 and dNd Scv) to identify the potential driver genes mutated in CRCOM (**Supplementary Data 4**), and summarized a list of 19 potential driver genes (including KRAS, TP53, APC, BRAF, RNF43, PCDHB12, ACVR2A, ZNF160, ZNF716, STOML1, SMIM3, NLGN1, DMD, LRP2, FAT4, ARID1A, NCOR1, RPTOR, SMAD3, MUC16). The well-known driver genes for CRC, such as TP53, NRAS, APC, and KRAS, were also mutated in our cohort. We also found that the mutation rate of several genes (including RPTOR, LRP2, NLGN1, and ZNF160) in OM was higher than that in primary CRC (**Figure 2C**).

The single-nucleotide variations (SNVs) displayed a preponderance of C > T transitions in primary CRC and OM. SNVs displayed considerable variations across and within patients, indicating intratumor heterogeneity. The SNV pattern in the P2, P3, P5, P6, P7, and P9, is similar between the primary and metastatic lesions, except for Patient 11. For P1 and P8 with bilateral ovarian metastasis, the SNV pattern is also highly similar between bilateral ovarian metastasis, but not similar in P4 and P10 (**Figure 2D**). The contributions of various known signatures to each sample are demonstrated in **Figure 2E** (**Supplementary Data 5**). Signature 1,6 and 30 were prevalent in CRC primary CRC and OM. Signature 4 was only prevalent in primary CRC, while Signature 3,11 and 12 were only prevalent in OM. Signature 3 was identified in 60% (9/15) of ovarian metastases, indicating that DNA double-strand break-repair (DSB) was highly involved in the etiology of CRCOM. To further determine the changes in Signature 3, we analyzed the DSB-related genes and found the mutation of USP7 (20% *vs* 9%), the rate of deletion in TP53BP1 (60 *vs* 45%), and RPA1 (93 *vs* 64%) were higher in OM (**Figure 2F**).

**Figure 3:**
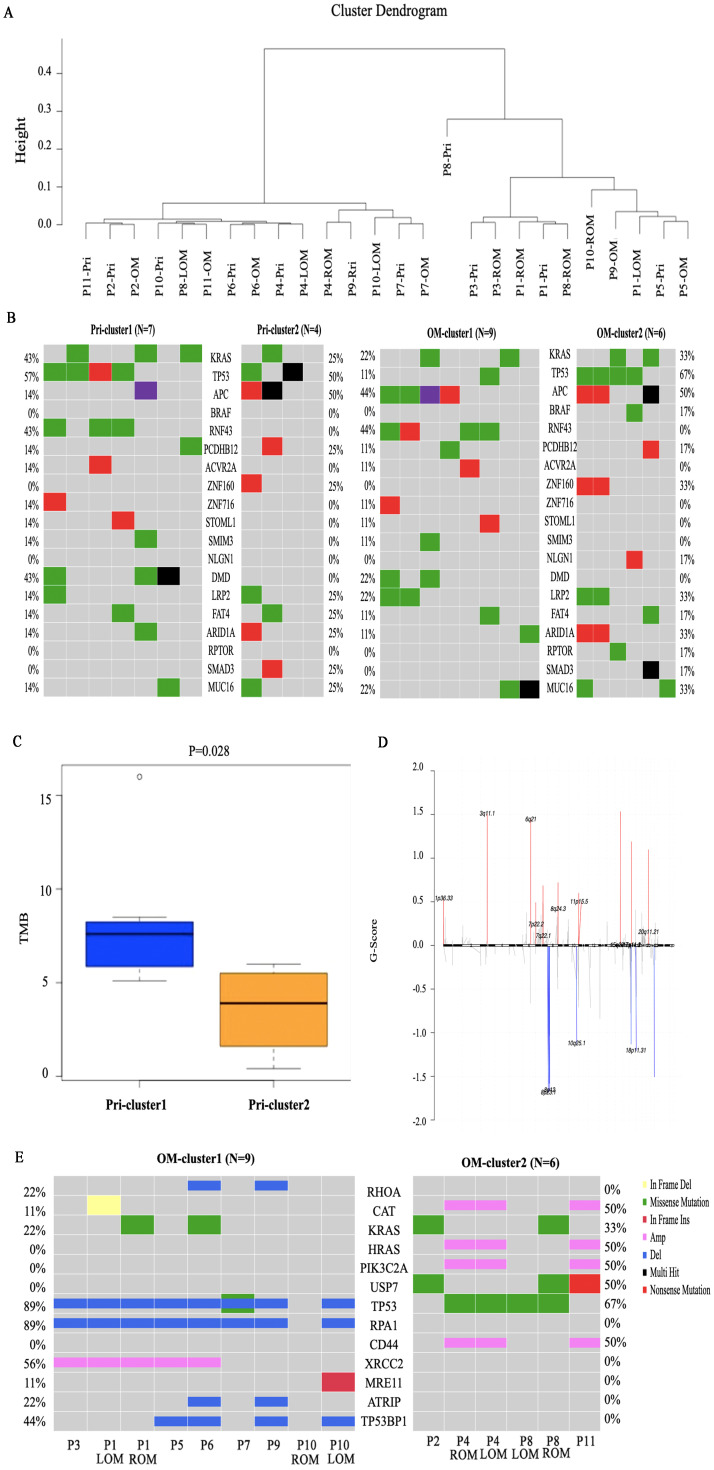
Genomic heterogeneity among different clusters. **(A)** The unsupervised clustering of all 11 patients is based on COSMIC Mutational Signatures. **(B)** The mutation of the list of 19 potential driver genes in primary CRC and OM of two clusters. **(C)** The tumor mutation burden (TMB) of primary CRC in two clusters. **(D)** The copy number alterations in OM of NMF_cos2. **(E)** The significantly different genes of OM between NMF_cos1 and NMF_cos2.

Based on the similarity between the COSMIC signatures features and the average link of the CRC primary lesions, non-negative matrix factorization (NMF) hierarchical clustering was performed on 11 patients. The patients were divided into two groups, the NMF_cos1 group including patient 1,3,5,6,7,9 and 10, as well as NMF_cos2 group including patient 2,4,8 and 11 (**Figure 3A**). Unsupervised clustering was also performed on all OMs, and patient 2,4,8 and 11 can also be clustered in one group (**Supplementary Figure 2A**). Survival analysis showed that there was a difference in overall survival (OS) between the two groups of patients (median OS, 720 days *vs* 360 days, *P* = 0.074) (**Supplementary Figure 2B**). To further explore the reason about CRC patients with OM in NMF_cos1 have better prognosis, we analyzed the genomic heterogeneity among different cluster samples. The known genes in CRC were frequently mutated both in primary CRC of NMF_cos1 or NMF_cos2, including KRAS, TP53, APC, PCDHB12, ZNF160, LRP2, FAT4, MUC16, and ARID1A, however, the mutation rates of these genes were different. As for OM, we found the rate of mutation of TP53 was higher in NMF_cos2. Besides, RNF43 and DMD are mutated only in primary CRC and OM of NMF_cos1 (**Figure 3B**). Significant heterogeneity was observed in two clusters since the median of tumor mutation burden (TMB) of primary CRC in NMF_cos 1 was 8.12/MB, which is greater than NMF_cos2 (3.55/MB, *P* = 0.028) (**Figure 3C**). The homologous recombination (HRD-score) was higher both in primary CRC and OM in NMF_cos1 than NMF_cos2 (**Supplementary Figure 2C**). The different SNVs and signatures are shown in (**Supplementary Figure 3**). To further determine the changes in genome segments of two clusters, we analyzed copy number alterations (CNA) in two clusters using Gistic 2.0. However, we don’t detect any significant CNA in NMF_cos1. The significant focal deletion of 17p11.2 and 18p11.31 are detected in all OM of NMF_cos2 (**Figure 3D**). We calculated the significantly different genes of OM between NMF_cos1 and NMF_cos2, and we found USP7 was significantly higher in NMF_cos2 (0 *vs* 3, *P* = 0.0439) (**Figure 3E**, **Supplementary Data 5**). We also collected the data of CRC patients in TCGA and found the mutation of USP7 is associated with poor DFS (**Supplementary Figure 2D**).

### 3.3 Clonal origin and spread of CRC with OM

**Figure 4:**
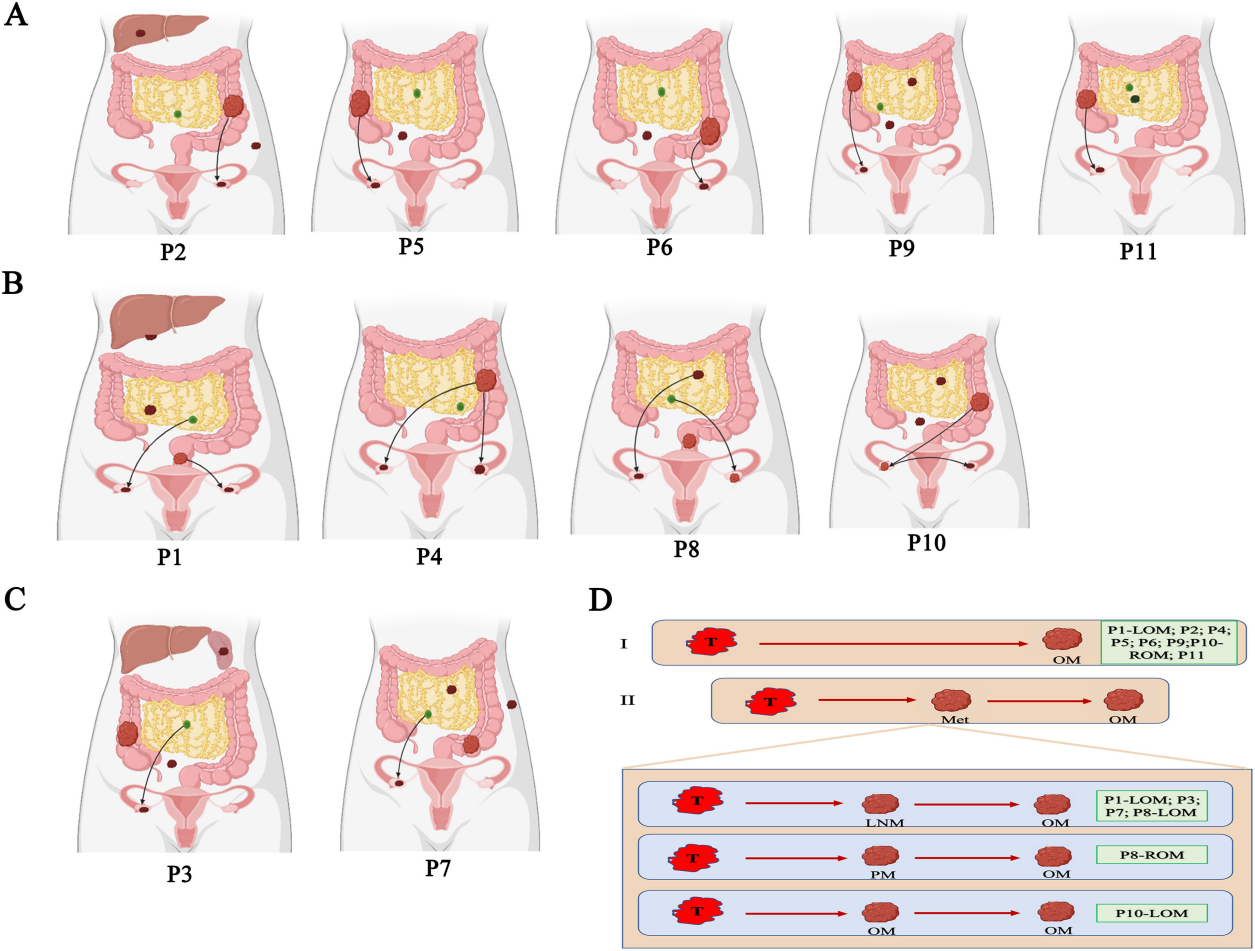
Parsimonious metastatic map and two modes of metastasis in CRCOM. **(A)** Primary CRC-seeding-OM models in CRCOM (including P2, P5, P6 and P9). **(B)** The models of bilateral OM (including P1, P4, P8 and P10). **(C)** LNM- seeding-OM models in CRCOM (including P3 and P7) **(D)** two modes of metastasis in CRCOM: primary CRC to OM and metastases to metastases (including LNM to OM, PM to OM, and OM to OM).

The main goal of our study is to illuminate the evolutionary relationship between primary CRC and OM. ClonEvol and MEGA 11 are used to build the phylogenetic tree of the CRCOM in each case (**Supplementary Figure 4**, **Supplementary Data 6**). We observed diverse evolutional patterns between primary CRC and OM. Firstly, OM derived from primary CRC is the main seeding model to describe metastasis dissemination. In P9, MEGA showed that the genetic distance of OM is closer to primary CRC. Similarly, ClonEvol showed that OM may come from primary CRC (**Figure 4**). The same phenomenon was found in P2, P4, P5, P6, and P11. Interestingly, there were bilateral OM in P4, however, the ROM and LOM are both seeded by primary CRC. Secondly, the lymphatic origin of CRCOM has been evidenced in ROM of P1, LOM of P8, P3, and P7. Thirdly, we first analyzed the metastatic pathway of P1, who harbored bilateral OMs, LNM, PM, and primary CRC. We portrayed the potential metastatic map of P1 and we speculated that the origin of LOM and ROM in P1 was different. LOM was directly derived from the primary CRC. However, ROM was derived from lymph nodes. The same phenomenon was found in P8 and P10, who have bilateral OMs derived from different organs. In P10, the genetic distance from LOM to ROM was shorter than the distance from primary CRC or other metastasis, so we speculate that ROM from primary CRC and LOM from ROM. MEGA 11 showed a long genetic distance exists between LOM and ROM. ROM is closer to omentum metastasis while LOM showed a closer genetic distance to primary CRC. Clonevol also showed that right ovary metastasis could come from omentum metastasis, while LOM derived from primary CRC.

### 3.4 Immunogenicity heterogeneity across and within individuals

**Figure 5:**
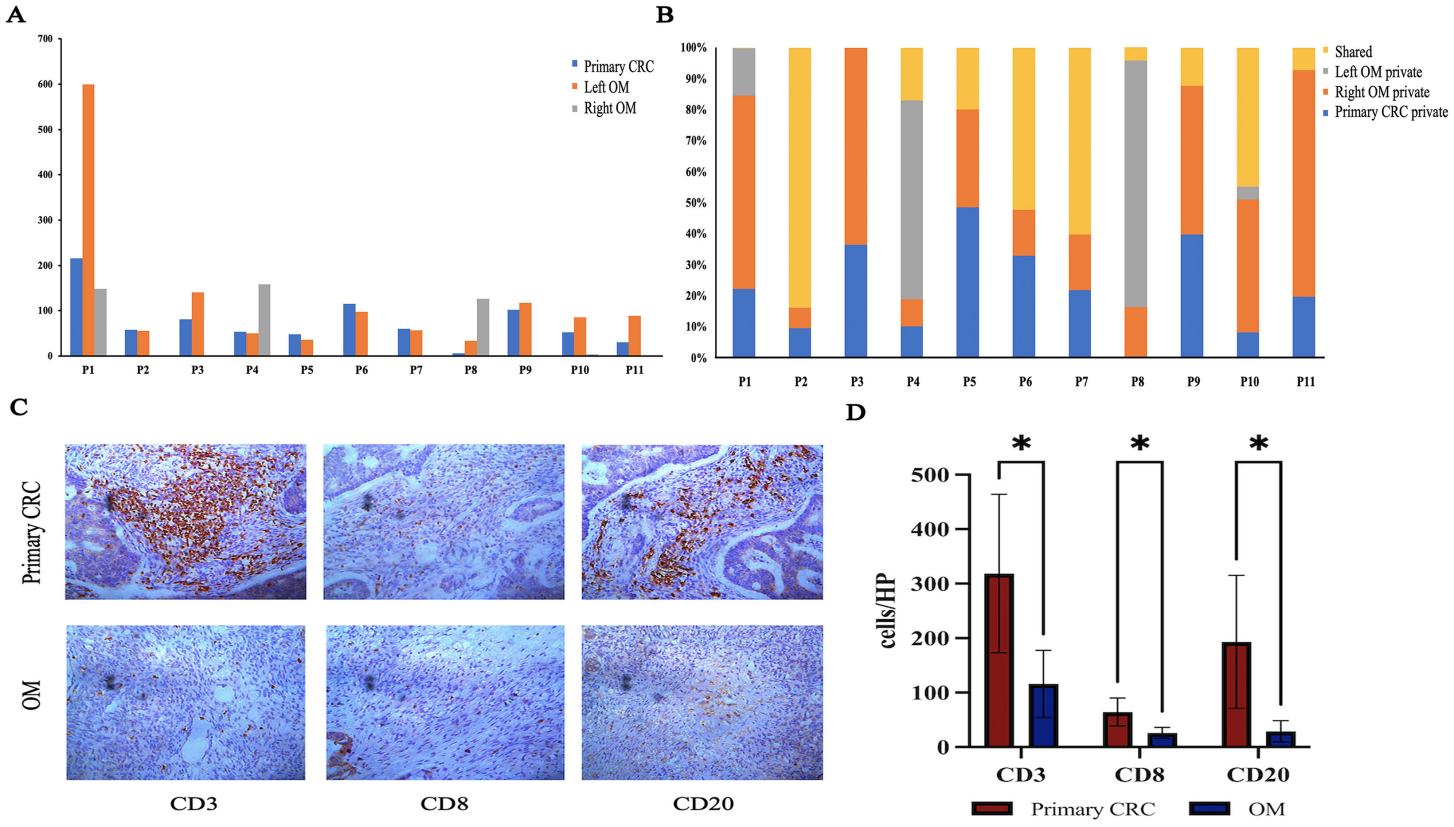
Immunogenicity heterogeneity across primary CRC and OM. **(A)** Predictive neoantigens numbers of CRC primary and OM. **(B)** Distributions of predicted neoantigens in each patient. **(C)** Image of primary CRC and OM tumor center with CD3+/CD8+/CD20+ staining. **(D)** The density was calculated as the number of positive cells/HP. (*represents P <.05).

Surgical resection and chemotherapy are the major choices for CRC patients with OM, immunotherapy is rarely applied in the treatment of CRCOM. We performed neoantigen number prediction to provide new insights into immunotherapy delivery in CRCOM. The predicted neoantigen number of each sample is shown in **Figure 5A**. The neoantigens of primary CRC and OM vary widely among patients, as well as a large difference in neoantigen between primary CRC and OM in the same patient. The predicted neoantigen number of primary CRC was higher than that of OM in NMF_cos1 patients. For NMF_cos2 patients (P2,4,8, and 11), the predicted neoantigen number of primary CRC and OM was lower than that of NMF_cos1 (P1, 3, 5, 6, 7, 9, and 10), suggesting neoantigens may be able to predict the infiltrating state and immune integral in tumor tissue (**Figure 5B**). The immunoscore is based on the infiltrating density of CD3^+^ and CD8^+^ TILs, and is used to predict the prognosis of patients with stage II and III colon cancer and has independent prognostic value. We compare the immune status of primary CRC and OM focus in 4 patients with CRCOM. The OM showed an immune desert state, extremely deficient in each subtype of immune cells (**Figure 5C**). Compared with the primary lesions, the infiltration of CD3+ T cells, CD8+ T cells, and CD20+ B cells, associated with better prognosis, were substantially lower in OM (**Figure 5E**).

## 4 Discussion

Although the diagnosis and treatment of metastatic CRC have been improved in recent years, CRCOM is still a big challenge for clinicians and cancer workers due to its special phenotype and unclear evolutionary relationship (36). Understanding the special phenotype and evolutionary relationship of CRCOM is important for the diagnosis and treatment of patients with CRCOM. Previous studies always explored the metastatic evolution in metastatic CRC by using single pairing (21, 22), however, it has been proved that metastatic CRC is a systemic disease with multi-organ involvement (37). In this research, we collected all the multi-site metastases in CRCOM patients, including OM, paired primary CRC, PM, LNM, LM, and so on. A total of 54 tumor samples and 11 normal tissues in 11 patients were collected to identify possible biological differences in OM, primary CRC, and other metastases, as well as portray a detailed metastatic map of CRCOM.

Based on our data, the significant genomic heterogeneity between primary CRC and OM has been evidenced. The most frequently mutated gene is TP53 in primary CRC while APC is in OM. Only 14/47 known driver genes were mutated in our study and 5 known driver genes were only mutated in OM, which implies CRC with OM may have unique mutation features. It will promote the progression of CRC due to the accumulation of mutations, which are the essential component of the signaling pathway in regulating cellular replication (38). SNVs displayed considerable variations across and within patients, also indicating intratumor heterogeneity. Previous research has shown the heterogeneity among metastases was minimal (39), however, we also found significant inter-metastatic heterogeneity between bilateral OM of P4 and P10 in our cohort, which might be explained by the multiple metastases rather than a single pairing in each patient. These results showed that the CRC cells must adapt molecular characteristics to escape from primary CRC and form CRCOM by interacting with tissue microenvironments across the ovary.

There is emerging evidence about the predictive role of homologous recombination deficiency (HRD) in multiple cancers (40). The mechanism of HRD is complex, as reflected by the variable definitions between studies. BRCA1/2 alterations are currently the main biomarkers of HRD (41). However, many tumors with phenotypic signatures consistent with HRD did not harbor BRCA1/2 mutations. There has been increased recognition of the role of other HRD-related mutations beyond BRCA and PALB2 and their potential to serve as predictive biomarkers (42). In this study, we found that signature 3 (associated with homologous recombination, HR) was identified in most CRC patients with OM, and the HRD-score was higher both in primary CRC and OM in NMF_cos1 than NMF_cos2. Cancers exhibiting HRD frequently demonstrate increased susceptibility to precision therapeutics, particularly poly-ADP-ribose polymerase inhibitors (PARPi). Given the restricted treatment alternatives for CRCOM, we will focus on investigating necessitate precision stratification strategies to delineate patient subgroups that may derive clinical benefit from PARPi, particularly those refractories to immune checkpoint blockade or conventional chemotherapeutic regimens. The mutation of USP7 and RPA1 is higher in OM. We divided patients into two groups according to NMF hierarchical clustering, the patients in NMF_cos1 have better prognosis than NMF_cos2. Comparing the two clusters, we also found the mutation of USP7 only existed in NMF-cluster 2. Ubiquitin-specific protease 7 (USP7) is one of the most abundant ubiquitin-specific proteases (USP), and plays multifaceted roles in many cellular events, including the p53-dependent DNA damage response (DDR) pathway (43, 44). USP7 is also a master regulator of genomic integrity pathways (45). Recent study showed USP7 deubiquitylates and stabilizes DDX3X, augments Wnt/β-catenin signaling, thereby facilitating CRC tumorigenesis (46). USP7 is also identified as a crucial role on YAP in the regulation of CRC cell proliferation and tumor growth (47). Yang et al. also found that STAT3 bound to the promoter region of USP7 and inhibited its activity through recruiting HDAC1. As a result of the decline of USP7 expression, endogenous P53 protein level was decreased (48). In CRC, USP7 also plays a key role in regulating YY1 protein levels, which promote tumor development. By binding to 296–414 amino acid residues of YY1, USP7 weakened its ubiquitination and degradation of K63 linkage, thereby extending the functional lifespan of YY1 (49). Recent studies have shown that USP7 deubiquitination and stabilization of β - catenin promote the occurrence of CRC (50). According to a meta-analysis, which had a total of 1192 patients and assessed five types of cancer, the high-expression of USP7 may promote the progression of epithelial ovarian cancer (EOC) and predict unfavorable prognosis of EOC patients (51). There are studies indicated that USP7 emerges as a potential therapeutic target for cancers, as it plays an important role in the development of tumorigenesis by stabilizing multiple cancer-relevant proteins. Selective USP7 inhibitor (e.g., N-benzylpiperidinol derivatives, erteporfin (VP), and Compound P5091) showed efficacy in CRC models (52) (48) (47). We found the deletion of 17p11.2 and 18p11.31 in all OM of NMF_cos2. Therefore, the subtypes of CRCOM with USP7 mutations and more copy number alterations had a worse prognosis. This evidence suggests that targeting USP7 may have therapeutic potential in CRC with OM. The prospective trials are needed to determine whether targeting HRD pathways (e.g., PARP inhibitors in USP7-mutant cases) or modulating the immunosuppressive microenvironment could improve outcomes. We propose a precision medicine framework where CRCOM molecular subtyping guides second-line therapy selection post-standard chemotherapy, pending validation in interventional studies.

Exploring the evolutionary relationship between primary CRC and OM is vital to choosing the best treatment for CRCOM patients. A notable finding is that we observed the models of evolution in primary CRC could impact the metastatic model. We observed that the metastases were seeded from multiple late subclones of primary CRC, resulting in inter-metastatic heterogeneity across metastatic lesions. Identifying these subclones with metastatic capacity could be helpful in early diagnosis and potentially curative treatment for CRCOM. According to the pattern of the metastatic pathway in each CRCOM patient, we summarized two different modes of CRCOM, including primary CRC to OM, and other metastasis to OM. Firstly, our data supported primary CRC invaded the ovary directly in most cases, according to CRCOM derived from primary CRC in 9/15 cases. Some studies showed that hematogenous pathways were vital in CRCOM because both primary CRC and ovary are rich in blood vessels with frequent cancer embolus (53, 54). Besides, CRCOM was usually detected in young women, whose ovulatory cycle provided a suitable microenvironment for CRC cells to survive and invade (55, 56). Secondly, based on our data, the lymphatic origin of CRCOM has been evidenced in 4 patients, cancer cells first spread to adjacent lymph nodes and then metastasized through the lymphatic system to the ovary. Lymphatic origin was the widely accepted model in the CRC distant metastasis pathway, the presence of LNM is an important prognostic factor for CRC patients based on this model. Previous studies have shown that CRCOM was an independent risk factor for retroperitoneal lymph node recurrence (*P* = 0.0012) (57). They reviewed 105 CRC patients with PM who underwent surgery and HIPEC, of whom 62 patients also had OM. Retroperitoneal lymph node recurrence in CRC patients after surgery is a rare phenomenon, which only occurs in about 1% of patients, however, 29% of CRCOM patients in that study (57, 58). Lymph node dissection during primary CRC surgery may help prevent CRCOM. Identifying the LNM with high metastatic potential is crucial for the diagnosis and treatment of CRCOM since not all LNMs have the same metastatic potential. Besides, there were two patients with bilateral CRCOM, however, the sources of bilateral CRCOM were different in each patient. Thirdly, the evolutional patterns of P1, P8, and P10 also supported a model of metastasis-seeding-metastasis. In P4, ROM and LOM are seeded by different subclones in primary CRC, supporting polyclonal metastasis existing in the primary-seeding-metastasis model. Branched evolution has classically been viewed as the predominant evolution model in the process of tumor dissemination. These results showed that CRCOM is a complex process that may require the cooperation of multiple cells from different subclones, or occur during continuous evolution involving different clones. In conclusion, these results indicated that there were multiple metastasis pathways in the same CRCOM patients. Cancer cells from both primary CRC and other metastases could metastasize to the ovary and then form OM, and primary CRC and LNM were the important sources of CRCOM. More experimental and clinical studies are needed to verify the specific metastatic pathway and mechanism of CRCOM and then to apply them in developing precision therapy.

We offered novel insights for the immunotherapy administration in CRC with OM. There is emerging evidence that immune checkpoint inhibitors achieved considerable success in multiple malignancies, but this is less defined in CRCOM. We also observed that the multiple tumors within individuals were highly heterogeneous in neoantigen, while disparities exist between primary CRC and OM. The immunoscore provides a reliable estimate of the risk of recurrence in patients with colon cancer. We assessed the immunoscore by quantifying the densities of CD3+ and cytotoxic CD8+ T cells in the tumor and in the invasive margin of patients with CRCOM and found the immunoscore of CRCOM is low. Our findings shed light on the application of ICIs (immune checkpoint inhibitors) on CRCOM and suggested that different strategies should be applied to primary CRC and OM. The selection of CD3, CD8, and CD20 was driven by their established prognostic value in CRC and technical feasibility for multi-sample cohort analysis (59, 60). These markers provide a foundational assessment of adaptive immune cell recruitment. While our study characterized the immune landscape using CD3, CD8, and CD20 as key markers for T-cell and B-cell infiltration, we recognize that additional markers (e.g., PD-1/PD-L1 for immune checkpoint activity, FOXP3 for Tregs, CD68/CD163 for macrophage polarization) are critical to fully dissect the immunosuppressive mechanisms in CRCOM. The absence of these analyses may limit our understanding of therapeutic vulnerabilities, such as potential responsiveness to immune checkpoint inhibitors.

This study has several limitations that warrant consideration. First, the small cohort size (n=11 patients, despite multi-site sampling of 65 tissues) may restrict the statistical power and generalizability of our findings, particularly for subgroup analyses such as bilateral ovarian metastases comparisons. Future validation in larger, independent cohorts is imperative to confirm the clinical relevance of the proposed molecular subtypes and metastatic patterns. Second, all samples were derived from a single tertiary hospital in China, which may introduce selection bias toward patients with specific clinical profiles and limit extrapolation to other populations or healthcare settings. We will recruit external validation using geographically diverse cohorts to assess the robustness of our observations in the future. Furthermore, the exclusively Chinese cohort raises concerns about genetic ancestry-specific effects, as known population differences in colorectal cancer driver mutations and immune microenvironment dynamics could influence CRCOM biology. Studies should include multi-ethnic cohorts to investigate potential ancestry-related differences in CRCOM biology and metastatic behavior in future. Lastly, the OMs were collected from secondary surgery in four patients, who have received adjuvant therapy. This might cause the accumulation of treatment-resistant mutations, however, previous research verified that adjuvant therapy didn’t affect building phylogenetic tree (37). Future multi-center studies with ethnically diverse cohorts, complemented by mechanistic validations, are essential to address these limitations and advance CRCOM precision medicine.

In conclusion, we described the special molecular features of CRCOM by comparing paired primary CRC and multi-metastases. Our data indicated that there was significant intertumoral heterogeneity among patients with CRCOM, besides intratumoral heterogeneity among primary CRC, OM, and other metastatic lesions. 19 genes were inferred as the potential driver genes of CRCOM. Moreover, the USP7 was identified as the prognosis biomarkers in CRCOM. The subtypes of CRCOM with USP7 mutation, more copy number alterations, lower neoantigens and immunoscore have a worse prognosis. We also portrayed two metastatic patterns of CRCOM: primary CRC to OM and metastases to metastases (including LNM to OM, PM to OM, and other metastases to OM), and LNM was one of the important sources of CRCOM. Biopsy and sequencing of CRCOM should be applied to understand the dynamics of cancer evolution and choose a better treatment to improve the clinical outcomes of patients with CRCOM.

## Data Availability

Publicly available datasets were analyzed in this study. This data can be found here: The raw sequence data reported in this paper have been deposited in the Genome Sequence Archive in the National Genomics Data Center, China National Center for Bioinformation/Beijing Institute of Genomics, Chinese Academy of Sciences, under accession number GVM000406 (Project: PRJCA011872).

## Supplementary material

Supplementary Figure 1 The mean tumor mutation burden (TMB) for primary tumors and ovarian metastases.

Supplementary Figure 2
**(A)** Unsupervised clustering was also performed on all OMs. **(B)** Survival analysis in overall survival (OS) between the two groups of patients. **(C)** The homologous recombination (HRD-score) of two groups. **(D)** Survival analysis of the mutation of USP7 in TCGA.

Supplementary Figure 3 The different SNVs and signatures of the two groups.

Supplementary Figure 4 ClonEvol and MEGA 11 are used to build the phylogenetic tree of the CRCOM in each case.
