# Supplementary figures and images for: The heterogeneity of genomic alterations, metastatic patterns and immune microenvironment in metastatic ovarian cancer originating from colorectal cancer
Source: Front Immunol. 2025 Jun 11;16:1593439. doi: 10.3389/fimmu.2025.1593439 (PMC12187686; doi:10.3389/fimmu.2025.1593439)

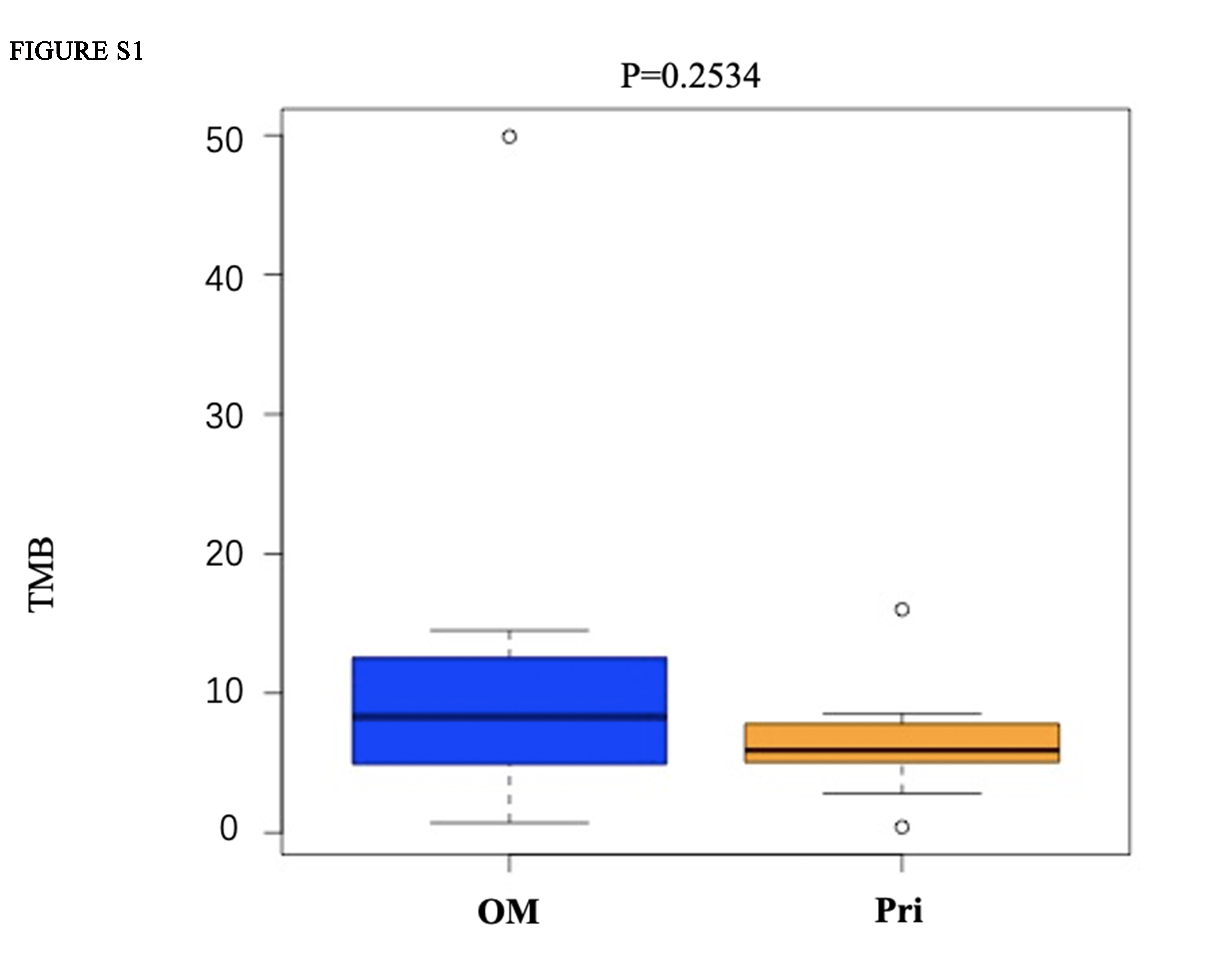

Supplement: Supplementary Figure 1 — The mean tumor mutation burden (TMB) for primary tumors and ovarian metastases. [file Image1.tif]

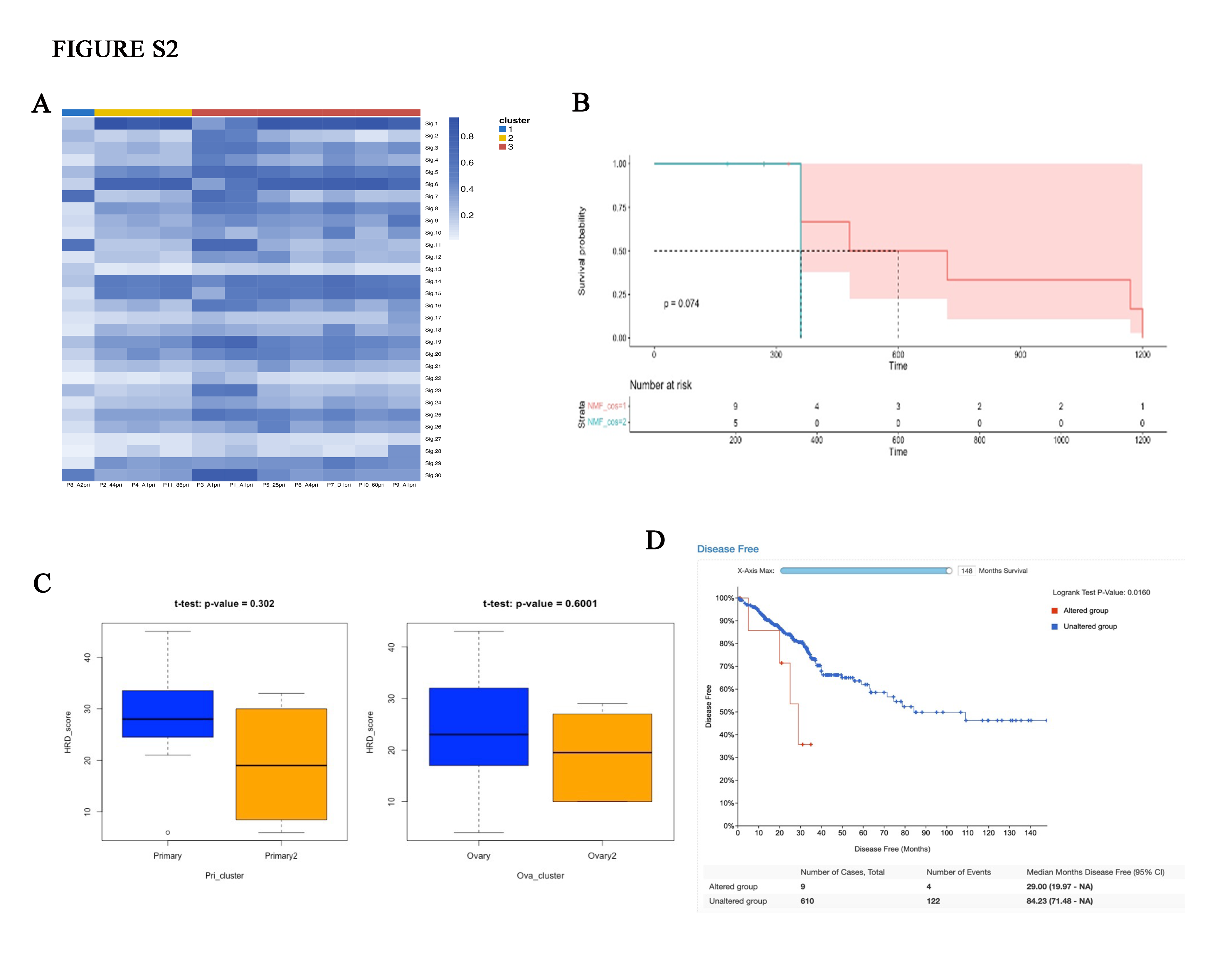

Supplement: Supplementary Figure 2 — (A) Unsupervised clustering was also performed on all OMs. (B) Survival analysis in overall survival (OS) between the two groups of patients. (C) The homologous recombination (HRD-score) of two groups. (D) Survival analysis of the mutation of USP7 in TCGA. [file Image2.tif]

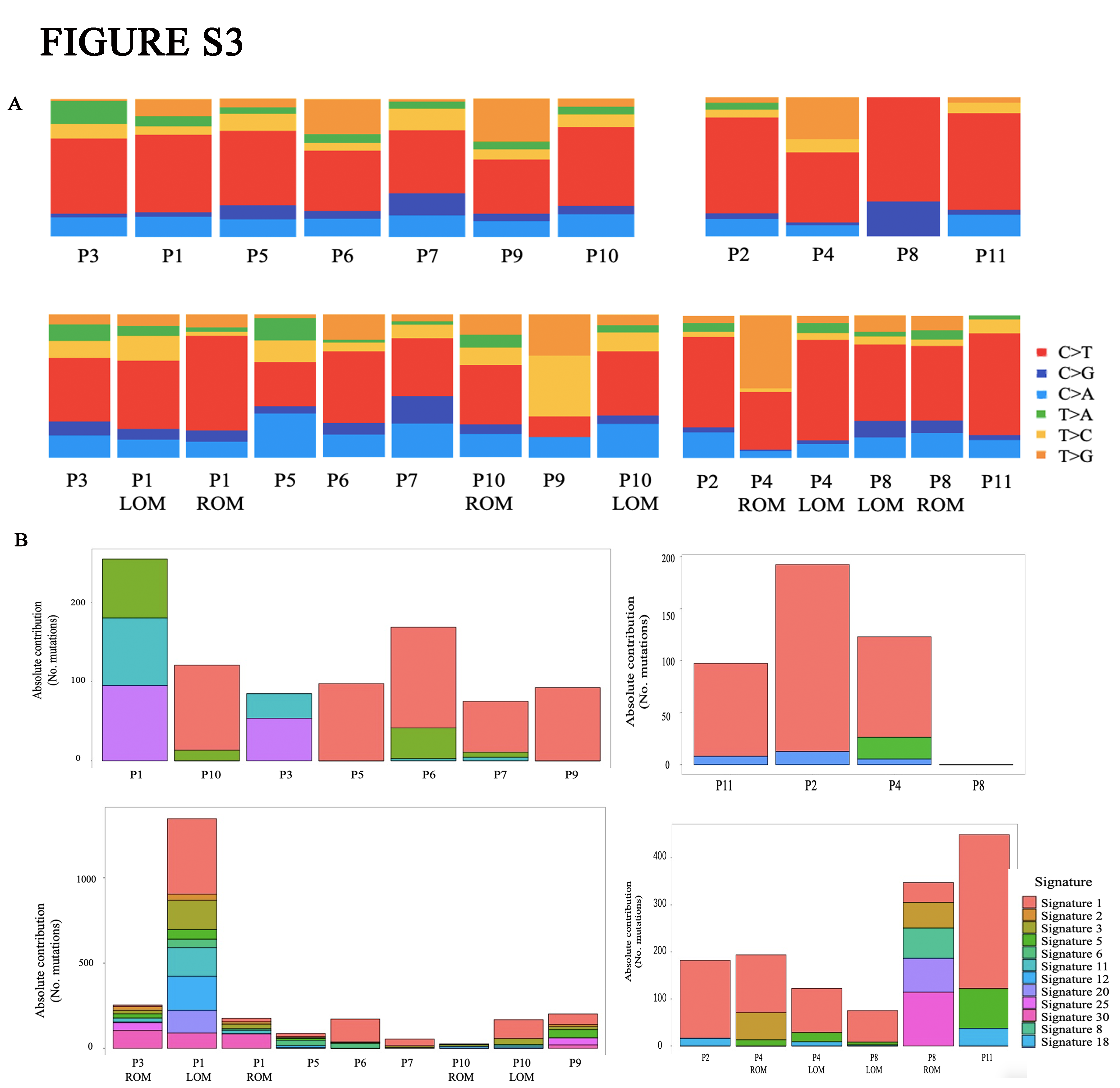

Supplement: Supplementary Figure 3 — The different SNVs and signatures of the two groups. [file Image3.tif]

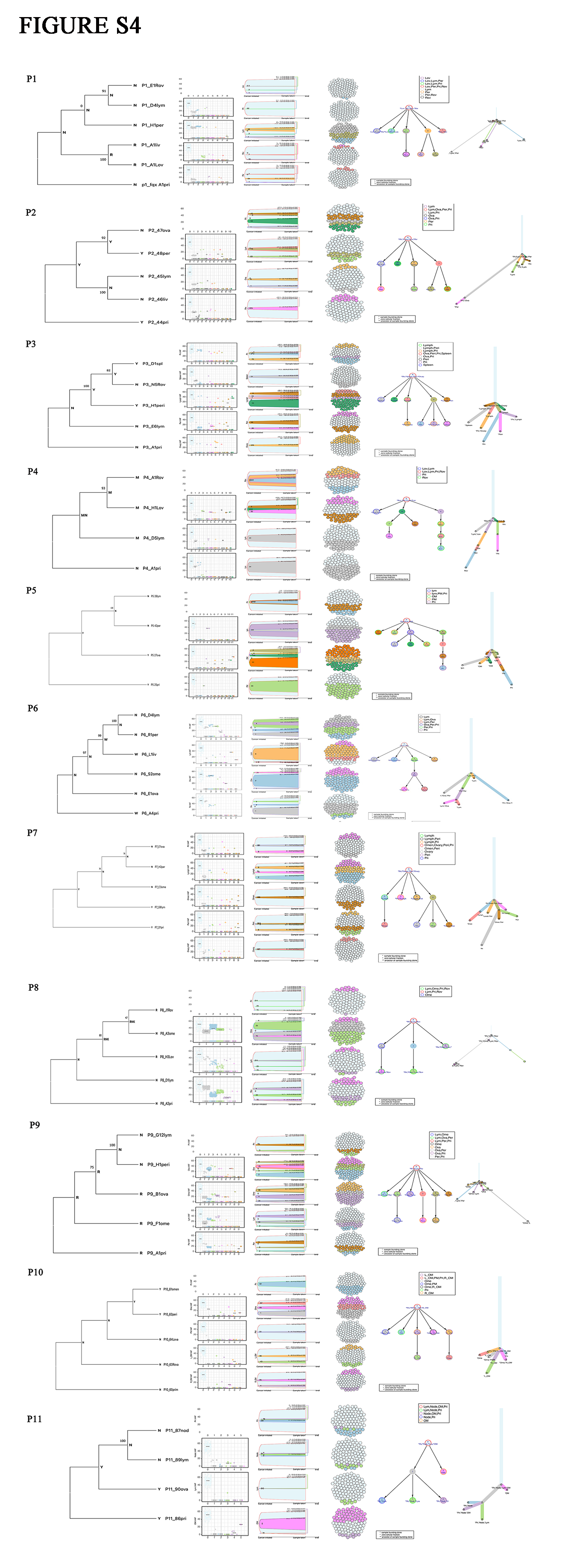

Supplement: Supplementary Figure 4 — ClonEvol and MEGA 11 are used to build the phylogenetic tree of the CRCOM in each case. [file Image4.tif]
